# Sex, Age, and BMI Modulate the Association of Physical Examinations and Blood Biochemistry Parameters and NAFLD: A Retrospective Study on 1994 Cases Observed at Shuguang Hospital, China

**DOI:** 10.1155/2019/1246518

**Published:** 2019-06-24

**Authors:** Zhengli Tang, Minh Pham, Yiming Hao, Fang Wang, Devashru Patel, Lori Jean-Baptiste, Lin Fan, Weijian Wang, Yiqin Wang, Feng Cheng

**Affiliations:** ^1^Shuguang Hospital Health Examination Center affiliated with Shanghai University of Traditional Chinese Medicine, Shanghai 201203, China; ^2^Department of Mathematics and Statistics, College of Arts and Sciences, University of South Florida, Tampa, FL 33620, USA; ^3^Shanghai Key Laboratory of Health Identification and Assessment/Laboratory of Traditional Chinese Medicine For Diagnostic Information, Shanghai University of Traditional Chinese Medicine, Shanghai 201203, China; ^4^College of Computing, Georgia Institute of Technology, Atlanta, GA 30332, USA; ^5^Biomedical Sciences Program, College of Arts and Sciences, University of South Florida, Tampa, FL 33620, USA; ^6^Department of Pharmaceutical Sciences, College of Pharmacy, University of South Florida, Tampa, FL 33612, USA

## Abstract

**Objective:**

Previous studies have shown that some metabolic risk factors are related to nonalcoholic fatty liver disease (NAFLD). This retrospective study was performed to investigate the associations between physical examinations and blood biochemistry parameters and NAFLD status and to identify possible risk factors of NAFLD.

**Methods:**

Study participants underwent general physical examinations, blood biochemistry, and abdominal ultrasound evaluations. In addition, data regarding sex, age, ethnicity, medical history, and alcohol consumption of participants were recorded. Among the study participants (N=1994), 57.8% were male, 41.2% over the age of 50, and 52.6% with BMI≥24. 986 patients had NAFLD and 1008 had no NAFLD. We used effect size analysis and logistic regression to determine which physical examinations and blood biochemistry parameters were significant for the association between these parameters and NAFLD status.

**Results:**

Both the effect size and logistic regression indicated that BMI, diastolic blood pressure (DBP), triglycerides (TG), and serum uric acid (SUA) show a significant association with NAFLD. Females are overall at a higher risk of NAFLD, but factors such as high BMI, DBP, TG, and SUA increase the associated risk for both sexes. Compared with males, females have a higher risk of NAFLD given that they are over 50, overweight and obese (BMI at or over 24), or have high SUA. In terms of age, people older than 50 with high SUA, and people younger than 50 with high DBP and low-density lipoprotein cholesterol (LDL-C) all increase the risk of NAFLD. For BMI, high DBP and low high-density lipoprotein cholesterol (HDL-C) are risk factors for NAFLD in overweight and obese people (BMI at or over 24), whereas in normal weight and underweight people (BMI under 24), elevated LDL-C increases the risk of NAFLD.

**Conclusions:**

Our results revealed sex, age, and BMI modulate the association of physical examinations and blood biochemistry parameters and NAFLD, which may facilitate the development of personalized early warning and prevention strategies of NAFLD for at-risk populations.

## 1. Introduction

Nonalcoholic fatty liver disease (NAFLD) is a multifactorial disease, which is influenced by genetic factors as well as diet, exercise, and lifestyle habits. NAFLD can increase the risk of other liver diseases including nonalcoholic steatohepatitis-cirrhosis (NASH-cirrhosis) and NASH-hepatocellular carcinoma (NASH-HCC) [1]. Recent studies also showed that NAFLD was associated with increased risk of cardiovascular disease, chronic kidney disease, and colorectal neoplasm [2–4].

In recent years, NAFLD is common not only in developed countries, but also in developing countries and is therefore a global, rather than regional, public health issue [5–7]. Therefore, greater significance is being placed on early diagnosis and treatment of NAFLD, which could prevent or diminish morbidity and mortality associated with NAFLD.

It is widely accepted that there is a bidirectional relationship between NAFLD and various components of metabolic syndrome, particularly hyperglycemia and hypertension [1]. Obesity, excessive intake of simple sugars, and physical inactivity are also considered to be the dominant risk factors of NAFLD [8, 9]. Previous studies have shown that some physical examinations and blood biochemistry parameters are associated with NAFLD [10–13]. However, confounding factors such as sex, age, and obesity status may affect the accuracy of association models because they are closely associated with NAFLD [14–16]. The risk factors of NAFLD may vary in female and male, as well as in different age or body mass index (BMI) groups [17–20]. In this paper, we not only investigated the association between general physical examinations and blood biochemistry parameters and NAFLD status, but also estimated the effects of sex, age, and BMI on the association. This study will help identify risk factors and lead to better NAFLD prediction models.

## 2. Materials and Methods

### 2.1. Data Source

Our data were collected from participants (between 18 and 87 years old) who went through physical examinations in the Shuguang Hospital affiliated with Shanghai University of Traditional Chinese Medicine from March 2017 to February 2018.

### 2.2. NAFLD Diagnosis and Criteria for Inclusion and Exclusion

The diagnosis of NAFLD was based on the presence of steatosis after excluding competing causes of steatogenic liver disease including

(a) Significant alcohol consumption (more than 7 standard alcoholic drinks/week (70 g ethanol) in women, more than 14 (140 g) in men)

(b) Viral hepatitis caused by hepatitis A virus, hepatitis B virus, hepatitis C virus, hepatitis D virus, and hepatitis E virus based on serum test

(c) Drug-induced liver disease, including herbal medicines and dietary supplements

(d) Autoimmune liver disease based on clinical manifestations, inquiries and consulting of medical history, and abdominal color ultrasound, including autoimmune hepatitis (3 subtypes), celiac disease, primary biliary cholangitis, and primary sclerosing cholangitis

(e) Diagnosis of metabolic liver disorders based on clinical manifestations, inquiries and consulting of medical history, including Wilson's disease, alpha-1-antitrypsin deficiency, hemochromatosis, glycogen storage disorders, cholesterol storage disorders, etc.

The criteria for NAFLD inclusion were established according to the practice guideline of the diagnosis and management of NAFLD [21].

### 2.3. Study Design and Data Collection

A retrospective study was performed to investigate the association of physical examinations and blood biochemistry parameters and NAFLD status in adults. All participants underwent physical examinations, blood biochemistry, and abdominal ultrasound evaluations. In addition, data on sex, age, ethnicity, medical history, and alcohol consumption were recorded.

Anthropometric data were measured using standard methods published by the World Health Organization [22]. The BMI was calculated as body weight divided by height squared (kg/m^2^). According to the criteria recommended by National Health and Family Planning Commission of PRC [23], BMI<18.5 refers to underweight, 18.5≤BMI<24 refers to normal weight, 24≤BMI<28 refers to overweight, and BMI≥28 refers to obese. Blood pressure was measured using a mercury sphygmomanometer in a seated position after a 5-minute rest and was recorded as the mean of two different measurements taken within a 1-minute interval. A fasting blood sample was collected from each participant via the antecubital vein in the morning. Glucose (including FPG and HbA1c), serum lipids (including TC, TG, LDL-C, and HDL-C), indicators of liver function (including ALT, AST, and *γ*-GT), and indicators of kidney function (including SUA, SCr, and eGFR) were measured in the hospital laboratory according to routine procedures. In the same day, the participants' condition of NAFLD was judged by abdominal ultrasound evaluations.

The data collected includes 1994 individuals, 1152 of whom are male, 842 are female, 821 are 50 years old or older, 1173 are less than 50 years old, 945 are with BMI under 24, and 1049 are with BMI at or over 24. 986 patients were diagnosed with NAFLD and 1008 without NAFLD as controls.

### 2.4. Statistical Analysis

We used logistic regression to determine risk factors for having fatty liver for all patients and for each stratum (female versus male, older than 50 versus less than 50, and normal weight & underweight versus overweight & obese). Logistic regression has two important assumptions, linear relationship between the log odds of the outcome and the predictors and no multicollinearity between predictors. We first plotted the log odds of the outcome against each of the predictors to verify the first assumption. To prevent multicollinearity in the model, we selected variables for the models using the following process. First, we calculate the Variance Inflation Factor (VIF) for each variable given all other variables in the model. Then, the variable with the highest VIF is discarded and we recalculate the VIFs. These two steps are repeated until all the VIFs are lower than 3. The factors with* p*-value lower than 0.01 are considered significant risk factors and are highlighted in the tables.

We performed the logistic regression models by using R version 3.5.0 and the VIF calculations were done with the “car” library.

### 2.5. Ethics Approval

The study was approved by the Ethics Committee of Shanghai University of Traditional Chinese Medicine and was performed in accordance with the Declaration of Helsinki. All the subjects signed informed consent forms verifying consent and compliance.

## 3. Results

### 3.1. Comparison of NAFLD vs. Controls

Table 1 is a summary of the physical examinations and blood biochemistry parameters and demographic data of the sample population. We compared the control group with the NAFLD group by quantifying the effect size (Cohen's d). Based on magnitudes of Cohen's d, these factors can be divided into three groups, large (Cohen's d≥0.8), medium (0.8>d≥0.5), and small (d<0.5) effect size. Only there is a large difference in BMI values between control and NAFLD groups. Some factors (such as DBP, TG, HDL-C, SUA, ALT, and *γ*-GT) show medium differences between these two groups.

### 3.2. The Logistic Regression Models for All Individuals

To identify possible risk factors of fatty liver, we also performed a logistic regression analysis to investigate the association between the fatty liver occurrence with demographic or physical examinations and blood biochemistry parameters for all 1994 individuals (Table 2). ALT, AST, and *γ*-GT were excluded from the model because the increase of these liver function parameters could be the results of NAFLD. As shown in the table, factors including BMI, DBP, TG, and SUA show a very significant association with the fatty liver occurrence (*P*<0.01), which is consistent with the effect size calculation. The signs of the coefficients show that an increase in BMI, DBP, TG, and SUA are associated with the occurrence of NAFLD.

*Sex Difference*. As shown in Table 2, the* p*-value and positive coefficient for sex indicate that females are more likely to have NAFLD than males. In addition, the risk factors of NAFLD may be different between male and female. To investigate the sex difference in NAFLD, two logistic regression models were performed for 1152 male and 842 females, respectively, which can reveal how the significance of certain factors changed in accordance with sex. The results were shown in Table 3. The coefficients and* p*-values of the models indicate that factors (BMI, DBP, and TG) which are significant (*P*<0.01) for men all tend to be significant for women as well. It is noted that SUA level shows significance in female (*P*=0.0002) instead of male (*P*=0.26).

*Age Difference*. Splitting the data into two different age groups can similarly show how factors are differently associated with NAFLD in the young (age<50) and the old group (age≥50).  Table 4 shows the logistic regression model results for 821 patients 50 years of age or older and 1173 patients under 50. As shown in Table 4, BMI and TG are common significant factors for the young and old groups. Younger patients benefit significantly from low DBP and LDL-C levels as well. However, DBP and LDL-C do not significantly affect older individuals. Instead, it should be noted that for patients over 50, one is significantly more likely to have NAFLD if they are female as well as have high SUA or eGFR.

*Obesity Effects*. Both effective size and logistic regression analysis indicate that BMI are significantly associated with NAFLD. To investigate the relationship between BMI and NAFLD, two separate logistic regression models were performed for normal weight and underweight (BMI under 24, n=945) and overweight and obese people (BMI at or over 24, n=1049). The results are summarized in Table 5. TG and SUA are common significant factors for the two groups. High levels of LDL-C are associated with the chance of NAFLD for those with a BMI under 24. Overweight and obese people are more likely to have the disease if they had high DBP, eGFR, or low HDL-C. They also tend to be more at risk if they are female.

## 4. Discussion

In this retrospective study, we collected physical examinations and blood biochemistry parameters, other factors such as sex, age, and obesity status, and investigated the associations between these factors and NAFLD. This is the first study to analyze the effect of sex, age, and BMI on the association between physical examinations and blood biochemistry parameters and NAFLD status. From the results, we observed the following important findings.

First, DBP and SUA are identified to be associated with NAFLD by both effect size (Table 1) and logistic regression (Table 2). And researchers also have observed that people with high blood pressure have higher risk of NAFLD [24, 25], which is consistent with our findings.

SUA level is clinically associated with many diseases including metabolic diseases [26]. Three meta-analyses showed that people in the highest level of SUA had an exacerbated risk of NAFLD occurrence and the increased risk is probably independent of conventional NAFLD risk factors [27–29]. In our results, SUA has significant association with NAFLD and especially for women. A similar study indicated that the independent effect of hyperuricemia on NAFLD was stronger in women than in men [30].

Second, we show here that sex is associated with NAFLD. Some researchers found that males are more susceptible to NAFLD [31, 32]. But in those studies, the male group was compared to premenopausal women [33], who have a high level of estrogen which protects them from NAFLD [34]. When age was considered in a South China study, the incidence rates of fatty liver disease in women over 50 years old are higher than that in men, because women are no longer protected by estrogen as they were advanced in age [35]. In our research, the logistic regression model showed that among the individuals over 50 years old, females have positive association (coefficient =0.20) with NAFLD instead of males (Table 4). This positive association is not observed in the individuals less than 50 years old. The phenomena can be explained by the weaker protective effect of estrogen in postmenopausal women. In addition, we found that obese women are at higher risk of NAFLD, which is in line with the conclusion of Bedossa's group [36] and Lonardo's group [37].

Third, it was shown that high DBP, SUA, and LDL-C have different effects on the risk of NAFLD in different age groups. We noted that among the individuals under 50 years old, high DBP increases the likelihood of having NAFLD. The same positive association towards NAFLD is seen in individuals over 50 years of age with high SUA. Another interesting finding is that high LDL-C is a risk factor for NAFLD among younger people (<50 years). These results have not been reported by any other researchers.

Finally, in our results, people are more likely to have NAFLD when they have higher BMI. This result is not affected by age and sex. Obesity is one of the risk factors for NAFLD which has reached an agreement. This is also consistent with the findings from both the elderly and the youth [38, 39].

Dyslipidemia represents a key factor in NAFLD [40]. In this paper, TG, LDL-C, and HDL-C are the risk factors of NAFLD. Individuals whose BMI is under 24 kg/m^2^ are at increased risk in parallel with increasing levels of LDL-C. Another study also showed that nonobese people with higher LDL-C level within the normal range had an increased cumulative incidence rate of NAFLD [41]. Our study also indicates that overweight and obese individuals with low HDL-C or high DBP have greater odds of having NAFLD compared to individuals whose BMI is under 24. Although the mechanism by which this occurs remains to be further explored, it suggests that obese people should pay more attention to the impact of changes in levels of HDL-C and DBP.

In addition, the results show that the correlation between eGFR and NAFLD is positive when people are older (over 50 years old) or overweight/obese (BMI at or over 24). However, on the one hand, we find that eGFR in NAFLD group has no significant difference compared with normal weight people. On the other hand, sex differences need to be taken into account when calculating the eGFR value, and the results of both male and female groups showed no significant correlation. Therefore, the interpretation of eGFR needs further verification.

There are some limitations in our research. First, some known risk factors of demographic data for NAFLD, such as dietary preferences, exercise habits, work types, and so on, were not collected, which limits a comprehensive assessment of the correlation between risk factors and NAFLD. Second, the severity of NAFLD is not classified, and therefore the impact of risk factors on NAFLD severity was unknown. Third, we did not use a liver biopsy for NAFLD diagnosis, although our noninvasive diagnostic method was more suitable for the survey methods applied here. Lastly, we are unable to determine causal relationships due to the observational nature of the study.

Nevertheless, our results show that sex, age, and BMI have significant effects on the association between the physical examinations and blood biochemistry parameters and NAFLD. Some of these effects are supported by current literature, while others are novel. Our research demonstrates several new populations that may be at risk for NAFLD including people older than 50 with high SUA and people younger than 50 with high DBP and LDL-C. High DBP and low HDL-C are risk factors for NAFLD in people whose BMI is at or over 24. Future prospective studies are needed to confirm these effects, which will facilitate the development of personalized early warning and prevention strategies for NAFLD.

## Figures and Tables

**Table 1:** Differences of demographic and physical examinations and blood biochemistry parameters between control (n=1008) and NAFLD (n=986) groups.

Parameters	Control group	NAFLD group	Effect Size (Cohen's d)
Sex, male (%)	48%	52%	N/A
Age (years)	45.61 ± 13.23	48.27 ± 11.47	0.21
BMI (kg/m^2^)	22.54 ± 2.58	26.31 ± 3.29	1.28
SBP (mmHg)	124.26 ± 18.35	133.11 ± 17.51	0.49
DBP (mmHg)	76.59 ± 10.65	83.35 ± 10.32	0.64
TC (mmol/L)	4.94 ± 0.9	5.16 ± 0.96	0.24
TG (mmol/L)	1.23 ± 0.75	2.12 ± 1.48	0.76
LDL-C (mmol/L)	2.82 ± 0.77	3.03 ± 0.78	0.27
HDL-C (mmol/L)	1.43 ± 0.34	1.23 ± 0.37	0.56
FPG (mmol/L)	5.11 ± 0.97	5.52 ± 1.24	0.37
HbA1c (%)	5.36 ± 0.72	5.62 ± 0.81	0.34
SUA (*μ*mol/L)	331.88 ± 86.09	382.42 ± 90.25	0.57
SCr (*μ*mol/L)	70.47 ± 15.85	72.69 ± 14.91	0.14
eGFR (mL/min)	103.17 ± 17.75	102.24 ± 17.42	0.05
ALT (U/L)	19.56 ± 12.7	31.09 ± 24.36	0.59
AST (U/L)	21.61 ± 9.31	25.24 ± 12.43	0.33
*γ*-GT (U/L)	25.24 ± 21.81	40.49 ± 34.64	0.53

**Table 2:** Results of the logistic regression model for all 1994 individuals.

	Coefficient	Standard error	*z* score	*p*-value
(Intercept)	-16.02	1.096	-14.611	< 2e-16
Age	0.009842	0.005973	1.648	0.09938
BMI	0.4155	0.02601	15.97	*< 2e-16*
SBP	-0.00839	0.004899	-1.712	0.08698
DBP	0.02882	0.008061	3.575	*0.00035*
TG	0.6537	0.0839	7.791	*6.63E-15*
LDL-C	0.1622	0.07728	2.099	0.03585
HDL-C	-0.3463	0.1919	-1.805	0.07111
SUA	0.002622	0.000854	3.072	*0.00213*
eGFR	0.009062	0.00399	2.271	0.02314
FPG	0.1848	0.08063	2.292	0.02189
HbA1c	0.01698	0.1127	0.151	0.88021
Sex, female	0.559	0.1498	3.732	*0.00019*

**Table 3:** Results of the logistic regression models for male (n=1152) and female (n=842) groups.

	Coefficient		Standard error		*z* score		*p*-value	
	Male	Female	Male	Female	Male	Female	Male	Female
(Intercept)	-13.2658	-20.2182	1.344677	1.951553	-9.865	-10.36	< 2e-16	< 2e-16
Age	0.002213	0.026611	0.006961	0.011978	0.318	2.222	0.7505	0.026312
BMI	0.355822	0.539703	0.032111	0.047346	11.081	11.399	*< 2e-16*	*< 2e-16*
SBP	-0.01002	-0.01074	0.006147	0.008632	-1.63	-1.244	0.10306	0.213405
DBP	0.027274	0.039274	0.010201	0.013805	2.673	2.845	*0.00751*	*0.004443*
TG	0.628818	0.76328	0.093718	0.188619	6.71	4.047	*1.95E-11*	*0.000052*
LDL-C	0.144987	0.101325	0.099366	0.129806	1.459	0.781	0.14453	0.435046
HDL-C	-0.35774	-0.13634	0.229178	0.347107	-1.561	-0.393	0.11853	0.694472
SUA	0.00116	0.00572	0.001034	0.001547	1.123	3.698	0.26159	*0.000218*
eGFR	0.011235	0.006441	0.005169	0.006578	2.174	0.979	0.02974	0.32745
FPG	0.183773	0.120944	0.103598	0.160726	1.774	0.752	0.07608	0.451758
HbA1c	-0.00308	-0.00211	0.161804	0.163338	-0.019	-0.013	0.98484	0.989681

**Table 4:** Results of the logistic regression models for young (age<50, n=1173) and old (age≥50, n=821) groups.

	Coefficient		Standard error		*z* score		*p*-value	
	< 50	≥ 50	< 50	≥ 50	< 50	≥ 50	< 50	≥ 50
(Intercept)	-15.9682	-15.3334	1.448125	1.603174	-11.03	-9.564	< 2e-16	< 2e-16
BMI	0.457926	0.384688	0.036495	0.03862	12.548	9.961	*< 2e-16*	*< 2e-16*
SBP	-0.0169	-0.00435	0.008363	0.006014	-2.021	-0.724	0.043331	0.46931
DBP	0.042735	0.01892	0.012281	0.011256	3.48	1.681	*0.000502*	0.09281
TG	0.564608	0.748372	0.106244	0.134605	5.314	5.56	*1.07E-07*	*2.7E-08*
LDL-C	0.288518	-0.05319	0.107188	0.115621	2.692	-0.46	*0.007109*	0.64548
HDL-C	-0.56439	0.032413	0.239389	0.297334	-2.358	0.109	0.018393	0.91319
SUA	0.001278	0.003663	0.001174	0.001254	1.089	2.921	0.27597	*0.00349*
eGFR	0.003757	0.016959	0.005241	0.005917	0.717	2.866	0.473422	*0.00415*
FPG	0.121241	0.222519	0.129608	0.104029	0.935	2.139	0.349559	0.03244
HbA1c	0.169614	-0.07565	0.170456	0.144627	0.995	-0.523	0.319707	0.60092
Sex, female	0.195866	0.945695	0.217201	0.214469	0.902	4.409	0.367179	*1.04E-05*

**Table 5:** Results of the logistic regression models for normal & underweight (BMI<24, n=945) and overweight & obese (BMI≥24, n=1049) groups.

	Coefficient		Standard error		*z* score		*p*-value	
	< 24	≥ 24	< 24	≥ 24	< 24	≥ 24	< 24	≥ 24
(Intercept)	-7.37867	-6.76135	1.383066	1.24913	-5.335	-5.413	9.55E-08	6.2E-08
Age	0.020527	-0.00213	0.008716	0.007568	2.355	-0.282	0.01852	0.77803
SBP	-0.00479	-0.00527	0.007151	0.006177	-0.669	-0.853	0.50343	0.39376
DBP	0.026534	0.042591	0.011578	0.010329	2.292	4.123	0.02192	*3.73E-05*
TG	0.721075	0.712001	0.120035	0.112809	6.007	6.312	*1.89E-09*	*2.76E-10*
LDL-C	0.332423	-0.02543	0.111361	0.102064	2.985	-0.249	*0.00283*	0.80321
HDL-C	-0.23655	-0.68309	0.241765	0.244448	-0.978	-2.794	0.32786	*0.0052*
SUA	0.003935	0.003483	0.001277	0.001066	3.082	3.267	*0.00205*	*0.00109*
eGFR	-0.00111	0.013969	0.005999	0.005162	-0.185	2.706	0.85311	*0.0068*
FPG	0.245978	0.084616	0.122277	0.122354	2.012	0.692	0.04426	0.48921
HbA1c	-0.08415	0.2245	0.185368	0.169064	-0.454	1.328	0.64984	0.18421
Sex, female	0.174253	1.036711	0.206224	0.207484	0.845	4.997	0.39813	*5.84E-07*

## Data Availability

The measurement data used to support the findings of this study are restricted by the Ethics Committee of Shanghai University of Traditional Chinese Medicine in order to protect patient privacy. Data are available from Zhengli Tang (Email: sgyytzl@163.com) for researchers who meet the criteria for access to confidential data.
